# Supplementary material for: Inhibitory Activity of Essential Oils of Mentha spicata and Eucalyptus globulus on Biofilms of Streptococcus mutans in an In Vitro Model
Source: Antibiotics (Basel). 2023 Feb 10;12(2):369. doi: 10.3390/antibiotics12020369 (PMC9952483; doi:10.3390/antibiotics12020369)
Supplement: Supplementary file 1 [file antibiotics-12-00369-s001.zip › antibiotics-2171049-supplementary.pdf]

Supplementary material

Inhibitory Activity of Essential Oils of *Mentha spicata* and *Eucalyptus globulus* on Biofilms of *Streptococcus mutans* in An In Vitro Model

Guillermo Ernesto Landeo-Villanueva [1], María Elena Salazar-Salvatierra [2], Julio Reynaldo Ruiz-Quiroz [2], Noemi Zuta-Arriola [3], Benjamín Jarama-Soto [4], and Oscar Herrera-Calderón [5, *], Josefa Bertha Pari-Olarte [6]; Eddie Loyo-la-Gonzales [7]

[1] Faculty of Pharmacy and Biochemistry, Universidad Nacional Mayor de San Marcos, Jr. Puno 1002, Lima 15001, Peru

[2] Institute for Research in Biological Chemistry, Microbiology and Biotechnology "Marco Antonio Garrido Malo", Faculty of Pharmacy and Biochemistry, Universidad Nacional Mayor de San Marcos. Lima Peru

[3] Faculty of Health Science, Universidad Nacional del Callao, Callao, Peru

[4] School of Human Medicine, Faculty of Health Sciences, Universidad Peruana Unión, Lima, Peru

[5] Department of Pharmacology, Bromatology and Toxicology, Faculty of Pharmacy and Biochemistry, Universidad Nacional Mayor de San Marcos, Jr. Puno 1002, Lima 15001, Peru

[6] Department of Pharmaceutical Chemistry, Faculty of Pharmacy and Biochemistry, Universidad Nacional San Luis Gonzaga, Ica 11001, Peru.

[7] Department of Pharmaceutical Science, Faculty of Pharmacy and Biochemistry, Universidad Nacional San Luis Gonzaga, Ica 11001, Peru

- Correspondence: oherreraca@unmsm.edu.pe; Tel.: +51 956550510

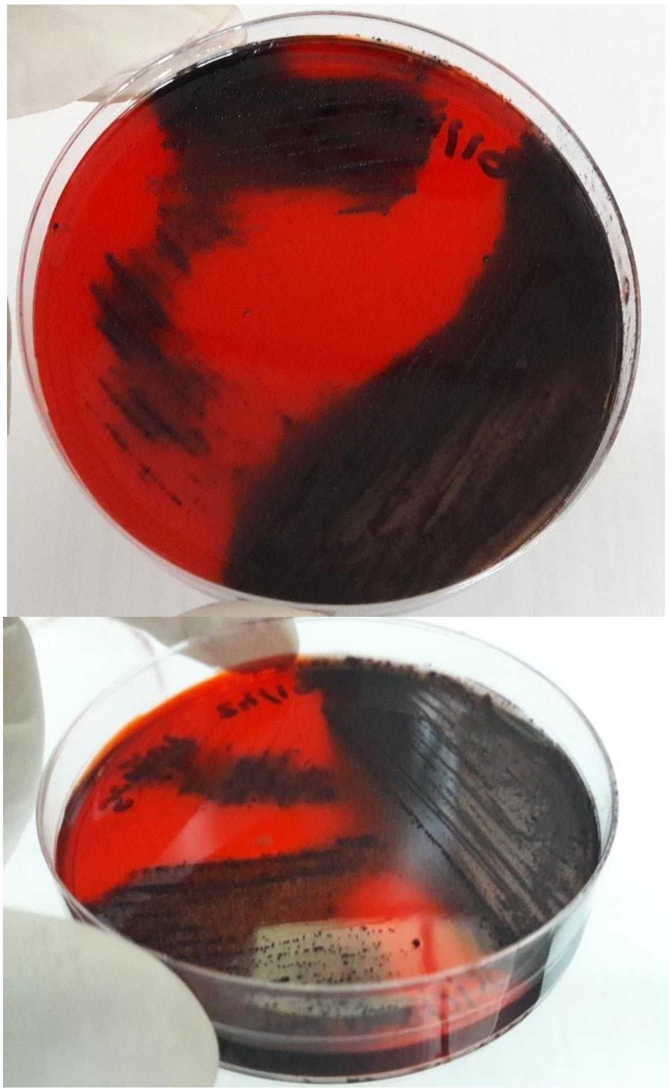

Figure S1: Ability to form biofilms by the Congo Red method.
